# Mutational load causes stochastic evolutionary outcomes in acute RNA viral infection

**DOI:** 10.1093/ve/vez008

**Published:** 2019-04-22

**Authors:** Lei Zhao, Ali B Abbasi, Christopher J R Illingworth

**Affiliations:** 1 Department of Genetics, University of Cambridge, Cambridge, UK; 2 Department of Applied Mathematics and Theoretical Physics, University of Cambridge, Cambridge, UK

**Keywords:** mutational load, within-host evolution, stochastic evolutionary processes, effective selection, acute viral infection

## Abstract

Mutational load is known to be of importance for the evolution of RNA viruses, the combination of a high mutation rate and large population size leading to an accumulation of deleterious mutations. However, while the effects of mutational load on global viral populations have been considered, its quantitative effects at the within-host scale of infection are less well understood. We here show that even on the rapid timescale of acute disease, mutational load has an effect on within-host viral adaptation, reducing the effective selection acting upon beneficial variants by ∼10 per cent. Furthermore, mutational load induces considerable stochasticity in the pattern of evolution, causing a more than five-fold uncertainty in the effective fitness of a transmitted beneficial variant. Our work aims to bridge the gap between classic models from population genetic theory and the biology of viral infection. In an advance on some previous models of mutational load, we replace the assumption of a constant variant fitness cost with an experimentally-derived distribution of fitness effects. Expanding previous frameworks for evolutionary simulation, we introduce the Wright-Fisher model with continuous mutation, which describes a continuum of possible modes of replication within a cell. Our results advance our understanding of adaptation in the context of strong selection and a high mutation rate. Despite viral populations having large absolute sizes, critical events in viral adaptation, including antigenic drift and the onset of drug resistance, arise through stochastic evolutionary processes.

## 1. Introduction

RNA viruses cause a broad range of acute infectious diseases. Seasonal influenza circulates as a global population, causing an estimated 3–5 million cases of severe illness each year (Bedford et al. 2015; WHO Influenza Factsheet 2018). Ebola virus generally causes small outbreaks, but from 2014 caused a severe epidemic in West Africa (WHO Ebola Response Team 2014). Rhinovirus, norovirus, measles, RSV, and parainfluenza all cause large numbers of infections each year. RNA viruses evolve on observable timescales (Biek et al. 2015); the potential to study evolution in this manner creates opportunities to combat viral disease.

Viral evolution acts across multiple scales, from the individual infection to the global evolution of viral populations (Murillo, Murillo, and Perelson 2013; Gog et al. 2015), with different factors driving evolution at different scales. Population bottlenecks occurring at transmission can cause the fixation of variants in a population by genetic drift (McCrone and Lauring 2018); larger population sizes during within-host infection can make selection the dominant evolutionary force (Pennings, Kryazhimskiy, and Wakeley 2014).

Here we consider viral evolution as it occurs within a single host. At the within-host level, a variety of studies have applied models to data, describing how selection shapes the genetic composition of a viral population over time (Ganusov et al. 2011; Kessinger, Perelson, and Neher 2013; Foll et al. 2014; Illingworth, Fischer, and Mustonen 2014; Illingworth 2015). Models used in this context typically assume either that alleles evolve in an independent manner, or that a few alleles, influenced by mutual linkage disequilibrium, evolve on an otherwise uniform genetic background. In either case the vast majority of loci in the viral genome are neglected. Such approaches allow for the rapid estimation of a fitness landscape, but neglect effects such as mutational load, which act in a truly genome-wide manner (Agrawal and Whitlock 2012).

The importance of mutational load for viral evolution is well-established (Pybus et al., 2007). RNA viruses exist at large within-host population sizes. Under high mutation rates viruses accumulate mutations, most of which are deleterious (Holland et al. 1982; Sanjuan et al. 2010). Experimental research has shown that tighter population bottlenecks accentuate the accumulation of deleterious mutations in viral populations (Elena et al. 2001; Miller, Joyce, and Wichman 2011); viruses may have evolved robustness in order to counter this effect (van Nimwegen, Crutchfield, and Huynen 1999; Manrubia et al. 2005).

The effects of mutational load have been considered in studies of infectious disease at the level of global populations. One study of the circulating influenza A/H3N2 population inferred that deleterious variants can, via linkage disequilibrium, fix in the viral population (Illingworth and Mustonen 2012). A further study of this population showed that presence of deleterious mutations slows the antigenic evolution of this virus (Koelle and Rasmussen 2015), the genetic background upon which a mutation arises delaying the onset of beneficial mutations.

The potential importance of mutational load for within-host viral evolution has been highlighted by a study showing the mutation rate for influenza to be higher than previously thought (Pauly, Procario, and Lauring 2017); more mutation clearly implies more mutational load. Mutational load is exploited by antiviral approaches which seek to increase viral mutation rates, the induction of deleterious mutations reducing the fitness of the population as a whole. Both experimental (Loeb et al. 1999; Crotty, Cameron, and Andino 2001; Arias, Thorne, and Goodfellow 2014) and theoretical approaches (Bull, Sanjuán, and Wilke 2007; Martin and Gandon 2010; Matuszewski et al. 2017) have been used to explore this strategy.

Mutational load has been well studied in population genetics research (Haldane 1937; Muller, 1964; Kimura and Maruyama 1966; Matuszewski et al. 2017), albeit that models of mutational load generally make the assumption that mutations have a constant deleterious fitness effect (Haigh 1978; Lynch et al. 1993; Koelle and Rasmussen 2015). An approach of this type has considered the effect of mutational robustness of a viral population, defined in terms of the proportion of mutations which are deleterious, and the effect which deleterious mutations have upon viral fitness, upon the consequent diversity of that population (Lauring, Frydman, and Andino 2013; Stern et al. 2014). However, recent studies measuring fitness effects in *in vitro* viral populations (Acevedo, Brodsky, and Andino 2014; Visher et al. 2016), have shown a distribution of fitness effects far from this assumption: A substantial proportion of mutations are lethal, with other mutations having a broad range of fitness effects. A gap therefore exists between traditional population genetic models of mutational load and biological reality.

We here adopt a new modelling approach to evaluate the effect of mutational load in a realistic model of acute within-host RNA infection. We introduce an extension to the standard Wright-Fisher population genetic model (Tataru et al. 2017) so as to explore the role of the intra- and inter-cellular lifecycle of an RNA virus upon its evolution, as part of a simulation of complex fitness effects.

Our model shows that under the influence of mutational load beneficial mutations have smaller and more stochastic effects in viral populations than has previously been appreciated. In a viral population, beneficial mutations can include variants conferring increased protein stability, immune escape (Grenfell et al. 2004; Leslie et al. 2004), drug resistance (Clavel and Hance 2004; Foll et al. 2014), and the adaptation of a zoonotic virus to a human host (Brander and Walker 2003; Taubenberger and Kash 2010; Moncla et al. 2016). We here evaluate the consequences of mutational load for the onset of a beneficial variant in a viral population in cases where the beneficial variant arises via *de novo* mutation and where the variant is transmitted on one virus in a population founding infection. Although parameterised for influenza, the generality of our model leads to an improved understanding of multiple questions in viral evolution.

## 2. Methods

In order to evaluate the effect of mutational load we derived an evolutionary model describing within-host growth, based upon the known characteristics of influenza viral infection. Simulations conducted using this model gave an insight into the behaviour of the system under a variety of evolutionary parameters.

### 2.1 Modelling framework

Previous models of within-host viral growth have considered the viral population either explicitly, accounting for each individual virus in the host (Russell et al. 2012) or implicitly, considering changes in the relative number of viruses over time (Beauchemin and Handel 2011). We here took the former approach, wishing to account for the fitness of each of the viruses in the system.

**Figure 1.**
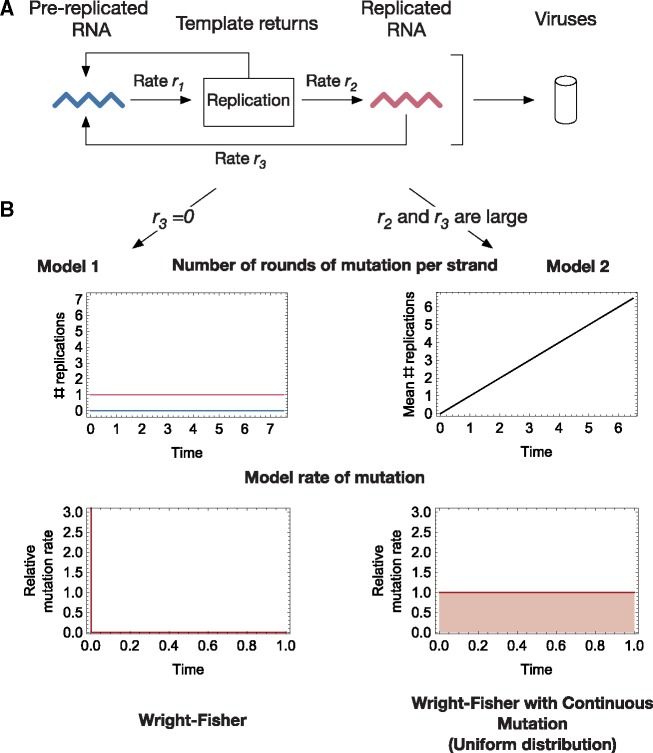
(A) Toy model of viral RNA replication. Following infection, strands of viral RNA enter the replication process at some rate *r*_1_. In replication a (mutated) copy of the original RNA strand is produced, the original immediately returning to the pool. Completion of the new RNA strand occurs at some rate *r*_2_, following which it is returned at some rate *r*_3_ to a state in which it can itself be replicated. RNA is translated to produce viral proteins; we assume that the amount of replicated RNA far exceeds the amount which enters the cell. (B) Consequences of specific model assumptions. If replicated RNA can never be re-replicated, all of the replicated RNA is copied from the genetic material of the viruses that founded infection; the vast majority of viral protein is therefore translated from RNA that has undergone a single round of mutation-prone replication. This can be represented by a model in which all of the mutation affecting the RNA forming the next generation of viruses occurs at a single time-point; in this case, mutation is described by a delta function at time *t* = 0. In contrast, if replicated RNA is completed and feeds back instantaneously, viruses translated from the RNA become progressively more mutated over time. This can be approximated by a model in which viral RNA acquires mutations at a constant rate.

The Wright-Fisher framework provides a computationally efficient description of an evolving population, being built upon the assumption that each generation of individuals arises from the reproduction of individuals in the previous generation. However, the reality of viral replication can be complex (Heldt, Frensing, and Reichl 2012). In order to think more deeply about how to model viral evolution we constructed a toy replication model (Fig. 1). Within a cell, viral RNA is replicated. New viruses are formed of proteins which have been translated from viral RNA; we assumed this to occur at a constant rate (Heldt, Frensing, and Reichl 2012). During replication the initial strand, once copied, is rapidly returned to the viral population. Copying is error-prone, with the copied strand being an imperfect replica of the original. The copied strand, which may take time to become fully formed, may or may not then feedback, in order to initiate further replication events. We note that the evolutionary dynamics of the system depend upon whether or not, and at what rate, replicated viral RNA feeds back into the viral replication process. If this feedback does not occur at all, the viruses produced by the cell are translated from viral RNA that, with the exception of the RNA which caused infection, has undergone a single round of error-prone replication. This process is well approximated by a Wright-Fisher model, in which the viral population undergoes a single round of instantaneous mutation before being subject to selection.

In contrast to this situation, a Wright-Fisher model is not fully accurate if replicated viral RNA is fed back in to the replication process. Considering the extreme case, in which feedback occurs instantaneously, we derived an alternative model. Under these circumstances, viral RNA is copied repeatedly over time, gradually acquiring mutations. Proteins translated from RNA early in this process thus have fewer mutations than proteins translated later in time. In Supplementary Material, we show that under an assumption of continuous protein production the acquisition of mutations per strand is roughly linear in time. The evolution of the system can in this case be represented by what we term a Wright-Fisher model with Continuous Mutation (WF-CM model), and specifically by a version of that model in which the timing of mutations follows a uniform distribution. We investigate both models, making the assumption that the biological reality is likely intermediate to the two cases we describe.

#### 2.1.1 Wright-Fisher model

In the Wright-Fisher model the population is evaluated at a set of discrete generations. We denote the fitness of the virus *i* as *w_i_*. In each generation each virus receives a Poisson-distributed number of mutations, with rate *μL*, where *μ* is the mutation rate of the virus and *L* is the length of the genome. The next generation of the population is then sampled from the current one; the probability that an individual in the next generation is descended from virus *i* is given by

(1) $$\frac{w_{i}}{\sum_{a}W_{a}}$$

where the sum is taken over all viruses *a*.

#### 2.1.2 Wright-Fisher model with continuous mutation

The WF-CM model is identical to the Wright-Fisher model, except that mutation is modelled as happening continuously throughout the process. Mutation occurs concurrent with the production of the next generation, with viruses that are produced earlier in the process carrying fewer mutations. Similar to the Wright-Fisher model, in each generation each virus receives a Poisson-distributed number of mutations, with rate *μL*. However, each mutation is in addition assigned a random time t∈[0,1].

**Figure 2.**
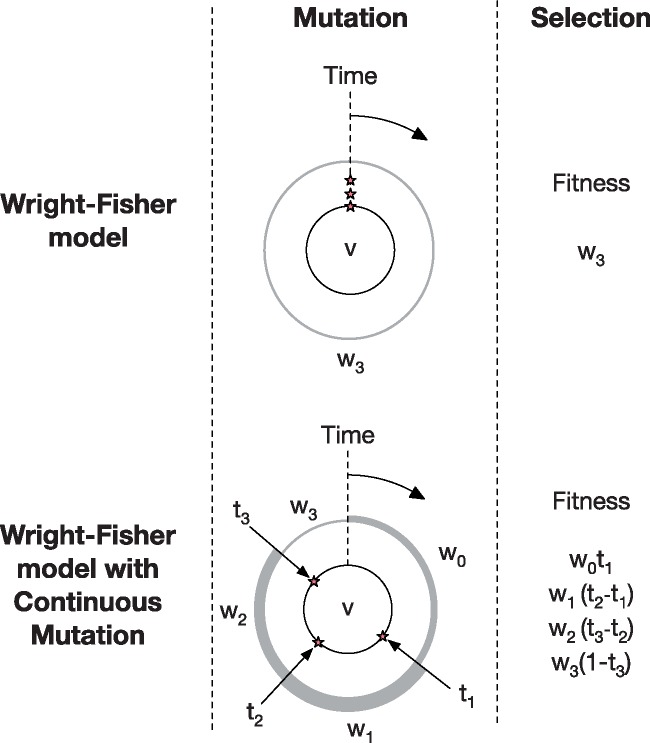
Illustration of the Wright-Fisher and WF-CM. In the example shown here, we assume that in the Wright-Fisher model a virus *v*, with original fitness *w*_0_, acquires three mutations in the mutation step, such that its offspring have the fitness *w*_3_; in the selection step these potential offspring compete with those of other viruses in the population. With continuous mutation these mutations arise at some times *t*_1_, *t*_2_, and *t*_3_ leading to the expression, during a generation, of potential offspring with fitnesses *w*_0_, *w*_1_, *w*_2_, and *w*_3_, indicated by the thickness of the different grey lines. In the selection step these potential viruses have fitnesses scaled by the length of time for which they are actively being produced.

Times assigned to mutations affect the manner in which the next generation is chosen. In the sampling step, suppose that in a given generation the virus *i* acquires *k* distinct mutations at the times $t_{1},t_{2},\ldots ,t_{k}$. We denote $t_{0}=0$ and $t_{k+1}=1$. The virus is then considered as *k* + 1 discrete objects $v_{i,0},v_{i,1},\ldots ,v_{i,k}$, where $v_{i,j}$ represents viruses descended from *i* during the time in which it has accumulated precisely *j* mutations. The virus exists in this state and produces offspring for a length of time $t_{j+1}-t_{j}$. Thus where $w_{i,j}$ is the fitness of *i* after it has accumulated precisely *j* mutations, the probability that an individual in the next generation is descended from $v_{i,j}$ is given by

(2) $$\frac{w_{i,j}(t_{j+1}-t_{j})}{\sum_{a}\sum_{b}w_{a,b}(t_{b+1}-t_{b})}$$

where the sum is taken over all *a*, *b*. In our model the times of mutation are chosen from the uniform distribution U[0,1]. This is not a rigid property of the model, but is derived from our toy model; other distributions of time could potentially be explored. A simple overview of the two models is shown in Fig. 2.

### 2.2 Mutation rate

The default mutation rate for our model was taken from recent experimental measurements conducted for the influenza A viral strain (Pauly, Procario, and Lauring 2017), which estimated a value of $\mu =1.8\times 10^{-4}$ per base per cycle of cell infection, somewhat higher than previous estimates (Nobusawa and Sato 2006; Sanjuan et al. 2010). We used this value as the default rate per base per generation in our model.

### 2.3 Distribution of fitness effects

We considered the fate of a single beneficial allele in the presence or absence of mutational load. The magnitude of selection for the single allele was set within a range making the variant advantageous enough to have some chance of being observed during the course of a single infection, yet not so advantageous that the fixation of the variant was inevitable. The fitness benefit conferred by the single allele was denoted as *s*, such that the allele granted a (1+*s*)-fold increase in the viral replication rate.

**Figure 3.**
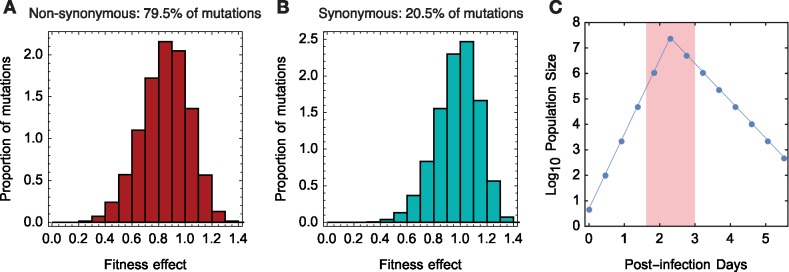
Inferred distributions of fitness effects for (A) non-synonymous and (B) synonymous variants. The non-lethal component of the non-synonymous distribution is Weibull-distributed with shape parameter 5.088 and scale parameter 0.8705. The synonymous distribution is Weibull-distributed with shape parameter 6.723 and scale parameter 0.9932. (C) Demographic model used in simulations, with an initial transmission bottleneck of 5 being simulated. The peak population size is close to 2.5 × 10^7^. Where the transmission of viruses was considered, it was modelled as occurring at a uniform time within the window of peak viral load, shaded in red. Alternative demographic models were defined as linear scalings of this model, defined by the population bottleneck incurred at transmission.

The distribution of fitness effects for other variants was set using data from *in vitro* experiments conducted with an influenza virus (Visher et al. 2016). This study measured the fitness effects of a set of synonymous and non-synonymous variants; ∼35 per cent of non-synonymous mutations were reported to be lethal, with no lethal synonymous mutations being found. Retaining these proportions of lethal mutations we fitted distributions to the data describing non-lethal mutations. A Weibull distribution was found to produce the best fit to the synonymous and non-synonymous mutation data if the fitted distribution was required to include both beneficial and deleterious effects (Table 1). Beneficial mutations were included in our fitness model to satisfy the property that influenza virus populations, which contain fixed deleterious variants (Illingworth and Mustonen 2012; Koelle and Rasmussen 2015), are unlikely to exist at a global fitness maximum. The fitted distributions are shown in Fig. 3A and B.

**Table 1.** Likelihoods for fitting a distribution of fitness effects to the experimental data.

Distribution	Exponential	Gamma	Weibull	Log-normal
BIC value	152.324	14.246	−24.208	32.386

A Weibull distribution is favoured under a comparison of models using the Bayesian information criterion (BIC). A lower value indicates a better model.

The fractions of synonymous and non-synonymous variants were calculated using the influenza A/Brisbane/59/2007 (H1N1) genome sequence. Variant types were calculated with reference to the proteins PB2, PB1, PB1-F2, PA, PA-X, HA, NP, NA, M1, M2, NS1, and NS2 reported in the NCBI influenza virus database (Bao et al. 2008). Where a variant caused different effect variants expressed in overlapping reading frames, the variant type was recorded as non-synonymous. The same reference sequence gave us a length for the genome of *L* = 13109 bases. We assumed a zero rate of within-host viral reassortment, matching the low rate observed in human infection (Sobel Leonard et al. 2017).

By default our simulations assumed a multiplicative model of fitness effects (e.g. two mutations which each reduce fitness to 90 per cent of its original value would together reduce viral fitness to 81 per cent of its original value). In some additional simulations negative epistasis was modelled as a constant negative effect applying in a pairwise manner between mutations. That is, if a virus with *k* variants had fitness *w* under the standard multiplicative model, its fitness given the influence of epistasis was equal to $w\chi^{k(k-1)/2}$, where *χ* is the effect of epistasis and the exponent term represents the number of pairs of variants in the viral sequence. The heuristic value χ=0.96 was used in our simulations, granting a weak epistatic effect between individual pairs, but contributing a stronger negative influence upon the accumulation of large numbers of mutations.

### 2.4 Demographic model

We conducted simulations under the assumption of a fixed demographic model; that is, where changes in the relative fitness of viruses do not affect the overall demography of infection. By default simulated infections were initiated with a founding population of five viruses, consistent with estimates obtained for RNA viral transmission (Zwart and Elena 2015). The population then followed a pattern of growth and decline matching that described by differential equation models of viral titre (Beauchemin and Handel 2011). In line with studies of single-cellular replication, the population increased twenty-two-fold in size each generation (Baccam et al., 2006) for five generations, before decreasing at a rate $\sqrt{22}$ per generation for a further seven generations (Fig. 3C). Informally a generation within our model corresponds to a period of just over 11 h (Baccam et al. 2006). Where a different founder population size was used, these relative changes in the size of the population were preserved, matching the experimental observation that an increased founder population causes a more severe infection (Graham and Braciale 1997).

### 2.5 Model implementation

Our evolutionary model requires the storage and evaluation of the fitness values of all individuals in the population, which becomes costly at large population sizes. To achieve this we discretised the space of possible fitness values into classes of size 0.01, with the fitness effects of new mutations being modelled as the transition of viruses between discrete fitness classes. Steps to speed up the computation of our simulations were taken, noting that as the population becomes large its behaviour becomes increasingly deterministic. Numerical approximations made in the model are detailed in Supplementary Material.

### 2.6 Equilibration of the model

Supposing the viral population to have been initiated by transmission, viruses in the founding population would likely differ with respect to the mutations they carry. To approximate the distribution of viral fitnesses of this population, simulations were run, representing the serial passaging of the virus through multiple hosts. Beginning from a homogeneous population, simulations of the growth of the virus under the influence of mutational load were conducted, taking an unbiased random sample from the population at a uniformly distributed time within the period of peak viral load. Repeated bottlenecks were calculated, allowing at least 100 generations of transmission for the equilibration of the distribution of viral fitnesses, following which sets of viral fitnesses in successive founder populations were collected; these fitnesses were used as starting points for further simulations. During this process the distribution of fitness effects was kept constant, fixations not inducing changes to this distribution. Distributions of relative viral fitnesses at the onset of infection, calculated for populations with a founder population of five viruses under different mutation rates, are shown in Supplementary Fig. S1.

### 2.7 Measuring effective selection

The effective selection acting upon a variant has been defined as the mean fitness advantage of individuals possessing the variant, accounting for linkage disequilibrium with other selected variants located elsewhere in the genome. It may differ from the inherent fitness acting for the variant in question (Illingworth and Mustonen 2011). We used successive frequencies of the selected variant from consecutive time points to measure the effective selection acting upon it. Denoting the frequency of the variant in generation *k* by $q(t_{k})$, we obtain for the Wright-Fisher model that

(3) $$s_{\text{eff}}=\frac{q(t_{k+1})-q(t_{k})(1-\mu )-\frac{\mu }{3}(1-q(t_{k}))}{(1-q(t_{k+1}))\left[q(t_{k})(1-\mu )+\frac{\mu }{3}(1-q(t_{k}))\right]}.$$

and for the WF-CM that

(4) $$s_{\text{eff}}=\frac{q(t_{k+1})-q(t_{k})(1-\frac{\mu }{2})-\frac{\mu }{6}(1-q(t_{k}))}{(1-q(t_{k+1}))\left[q(t_{k})(1-\frac{\mu }{2})+\frac{\mu }{6}(1-q(t_{k}))\right]}.$$

We note that the effective selection across a period of multiple generations can be easily calculated as the mean of $s^{\text{eff}}$ across the generations considered; when calculating this statistic from simulated data the calculation was performed over all generations for which the viral population size was equal to at least 10^4^ and for which $q(t_{k+1})$ was neither 1 nor 0. Derivations of Equations (3) and (4) are given in the Supplementary Mathematical Appendix.

Simulations were conducted using both the Wright-Fisher and WF-CM models. We considered both the case in which a beneficial mutation arose *de novo* within a viral population, and the case in which the beneficial mutation was carried by an individual in founder population, existing from the outset as standing variation.

## 3. Results

### 3.1 Variants arising through *de novo* mutation

#### 3.1.1 Mutational load reduces the effective selection acting for a beneficial variant

**Figure 4.**
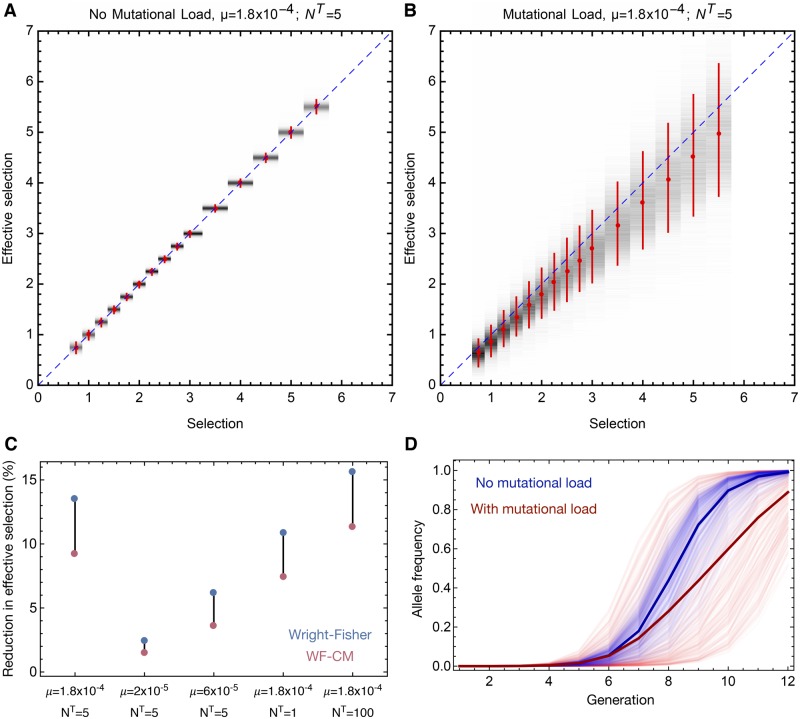
(A) Effective selection coefficients for beneficial mutations in simulations which excluded mutational load and were conducted using default model parameters. Red dots show the mean effective selection for mutations with a range of inherent selection coefficients. Vertical red bars show 90 per cent confidence intervals for the effective selection. Grey shading represents the distribution of inferred effective selection values. The blue dotted line shows equivalence between the true and effective selection coefficients. (B) Effective selection coefficients in simulations which included mutational load and were conducted using default model parameters. (C) Reduction in the mean effective selection coefficient relative to the inherent value of selection under different model parameters. Values are shown for simulations conducted with the Wright-Fisher (blue) and WF-CM (red) models. (D) Example trajectories for the beneficial variant under the WF-CM model with (red) and without (blue) mutational load. Selection here is equal to 3. Bold lines indicate mean trajectories. Trajectories shown here are those in which the beneficial variant is first observed in the third generation of the simulation.

Our simulations showed that mutational load reduced the mean effective selection acting for the beneficial variant, but greatly increased its variance. Where mutational load was not modelled in the simulation process, our estimates of the effective selection of this variant were almost exactly identical to the true selective advantage of the variant (Fig. 4A). Under these circumstances, the beneficial allele arises as an isolated variant on a uniform genetic background; minor deviations from its expected behaviour arise due to the effect of genetic drift on the variant while it exists at a low frequency. In contrast, where mutational load was incorporated into our simulation, the mean of the effective selection acting upon the variant was substantially lower than the simulated value, with a deficit of around 10 per cent on the inherent magnitude of selection being observed under default model parameters (Fig. 4B). This reduction in the effective fitness was close to being independent of the inherent benefit of the variant; a linear regression constrained to pass through the origin gave a value of *r*^2^ in excess of 0.999.

The reduction in the effective fitness can be understood in simple terms. Viruses possessing the beneficial variant can tolerate a greater number of deleterious mutations without becoming uncompetitive. As such, among viruses which are not eliminated by selection, mutational load has a greater deleterious effect upon viruses with the beneficial variant than upon those without it; the effective advantage of the beneficial variant is reduced.

The extent of the mean drop in effective selection varied according to the model used and its parameterisation. In our observations, mutational load had a lesser effect under the WF-CM model than under the standard Wright-Fisher population model (Fig. 4C). In the WF-CM model, mutations do not appear until some time into a generation. As such, the virus exists in its unmutated form for at least a short period before the arrival of the first mutation. Where mutations generally decrease viral fitness, this allows for the preservation of less-mutated viruses; fewer mutations are carried in the population in the WF-CM model than in the standard Wright-Fisher. This effect can be best appreciated in the case of lethal mutations; a virus which receives a lethal mutation may still produce offspring in the next generation; those offspring being produced in the fraction of a generation preceding the arrival of the lethal mutation. The WF-CM model with a uniform distribution of mutation times therefore provides a conservative estimate of the extent to which mutational load affects within-host evolution.

Example trajectories for the beneficial variant generated by the WF-CM model with and without mutational load are shown in Fig. 4D; an equivalent figure for the Wright-Fisher model is shown in Supplementary Fig. S2. Under our default parameters, the reduction in the mean effective selection was close to 9.2 per cent for the WF-CM model, and close to 13.5 per cent under the standard Wright-Fisher model.

Changes in the mutation rate of the model produced substantial changes in the mean influence of mutational load. Reducing the mutation rate of our model from 1.8 × 10^−4^ per base per generation to 6 × 10^−5^ per base per generation produced a reduction in effective selection of 3.5 per cent in the WF-CM model (6.1 per cent for the Wright-Fisher model), while a further reduction to 2 × 10^−5^ per base per generation produced a mean reduction of just 1.5% (2.4%); as would be expected a lowered mutation rate reduces the impact of mutational load.

Changes in our demographic model had smaller, but still measurable impacts on the effect of mutational load. Given a larger population bottleneck, a greater diversity of viral fitnesses exists in the population from the moment infection is initiated. In our approach a larger number of viruses founding infection corresponds to a larger viral population throughout the course of infection, such that the beneficial variant is likely to arise earlier in the course of infection. In our simulations a larger population led to a greater reduction in effective selection with a smaller bottleneck reducing the impact of mutational load. Given a bottleneck size of $N^{T}=1$, we observed a reduction of 7.4 per cent in effective selection acting on the variant under the WF-CM model (equivalently 10.8% in the Wright-Fisher model), while a bottleneck size of $N^{T}=100$ gave a reduction in the effective selection of 11.3 per cent (15.6%).

The addition of negative epistasis to the model led to a slight additional decrease in the effective selection of a variant. Although few empirical studies of patterns of epistasis in viral populations have been conducted (Lyons and Lauring 2018), the need for proteins to fold has been proposed to contribute a general pattern of negative epistasis between variant alleles (Sarkisyan et al. 2016; Wylie and Shakhnovich 2011). The addition of a small pairwise negative epistatic fitness cost to our model led to a further reduction in the absolute fitness of a beneficial allele, by a consistent amount close to 10.7 per cent of the original fitness of the variant (Supplementary Fig. S3).

Although mutational load decreased the mean effective fitness of a variant, the variance in that effective fitness of a *de novo* variant greatly increased under the influence of mutational load. Under our default parameters, the size of a confidence interval including 90 per cent of the effective selection coefficients tended to a range spanning values ∼25 per cent higher or lower than the mean effective value as the inherent fitness of a variant became large (Supplementary Fig. S4). At these high fitness values, viruses which gain the beneficial mutation become almost certain to produce offspring which survive until the end of the infection; the stochasticity in the effective selection arises from a combination of the set of genetic backgrounds upon which the variant can arise, plus the varied potential effect of subsequent mutations upon the system.

#### 3.1.2 Mutational load decreases the probability that a variant allele will emerge during the course of an infection

**Figure 5.**
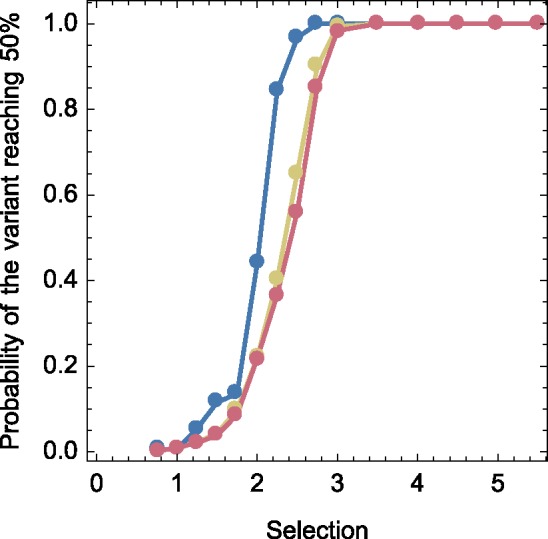
Probability that the beneficial variant will rise to a frequency of 50 per cent or greater during the course of an infection. Data are shown from simulations that exclude the effects of mutational load (blue) or include it, either under a multiplicative model of selection (yellow) or under a model incorporating negative epistasis (red). Simulations were conducted with an initial population bottleneck of 5 and a mutation rate of 1.8 × 10^−4^ per base per generation. Data are shown here for the WF-CM model.

The reduction in the mean effective fitness of a beneficial variant caused by mutational load in general reduces the probability of the variant emerging during the course of an infection. Here emergence was defined as the frequency of the beneficial mutation rising to 50 per cent or higher in the viral population by the end of infection. As shown in Fig. 5, this probability was almost universally reduced by the effects of mutational load. An exception to this occurs at the lowest selection coefficients considered. Although the mean effective selection is reduced by mutational load, the increased variance means that in some cases the effective fitness can lie above the inherent fitness of the beneficial allele. Where the inherent benefit is low enough that a mutation does not emerge in the absence of stochastic effects, mutational load led to a few cases in which emergence did occur. With this exception our result is straightforward; mutational load makes new alleles less likely to emerge during the course of an infection. Data shown here were generated using the WF-CM model; the Wright-Fisher model produced a slightly greater reduction in the probability of establishment (Supplementary Fig. S5).

### 3.2 Transmitted variants

We further considered the fate of beneficial variants that are transmitted from a previously infected host. Simulations were initiated describing populations in which one of the founder viruses in the population carried the beneficial mutation. In addition to measuring the effective selection acting upon the variant we measured the likelihood of its fixation (existing at a frequency ≥99 per cent by the end of an infection) or loss from the population (existing at a frequency ≤1 per cent by the end of the period of infection). We note that, due to the continual process of mutation, the absolute fixation or loss of variants is rare.

#### 3.2.1 Mutational load makes the fate of transmitted variants highly stochastic

**Figure 6.**
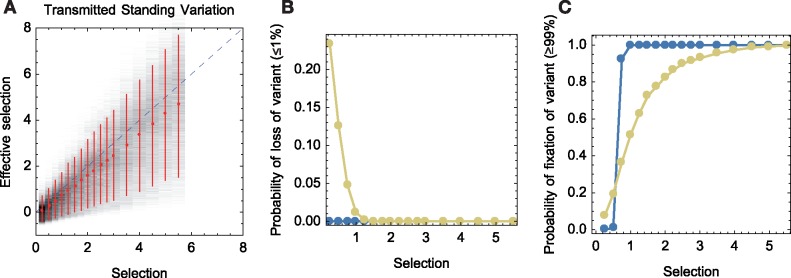
Effect of mutational load upon a transmitted variant. Simulations were initiated with one out of five viruses carrying the beneficial allele. (A) Effective selection coefficients for a beneficial mutation in simulations which included the effects of mutational load. Red dots show the mean effective selection for mutations of selection coefficients tested. Vertical red bars show 90 per cent confidence intervals for this statistic. Grey shading represents the distribution of inferred effective selection values. Simulations were conducted with an initial population bottleneck of 5 and a mutation rate of 1.8 × 10^−4^ per base per generation. (B) Probability that the beneficial variant will reach fixation during the course of an infection. Results are shown for simulations in the absence (blue) or presence (yellow) of mutational load. (C) Probability that a variant will die out during the course of infection. Error bars show the extent of variation across 10,000 simulations for each point. Data are shown here for the WF-CM model.

In comparison to the case of *de novo* mutation, a much greater variation in the effective fitness of the beneficial variant was seen in these simulations (Fig. 6A). At the largest inherent selection coefficients, the measured effective selection was between 30 and 165 per cent of the inherent value, a more than five-fold uncertainty in the effect of the variant; this range was larger at weaker magnitudes of selection. The increased variance arises to a small extent from genetic drift in the early phases of infection; while the viral population grows rapidly, the size of the population by the second generation is not large, at close to 100. Measurements of effective selection showed a variance of roughly ±20 per cent in the effective selection even where no mutational load was present (Supplementary Fig. S6).

The bulk of the increased variance in effective selection is therefore explained by effects arising from mutational load, specifically by variation in the fitness of the initial virus in which the beneficial variant appears. During early infection, the growth of the beneficial variant in the population arises from the clonal growth of the descendants of this single virus. Although new viruses carrying the variant arise *de novo* in later generations, this initial clade has a substantial impact upon the evolution of the population as a whole. The range in the potential fitness of a single transmitted virus is substantially larger than that in the multiple viruses upon which the beneficial variant arises in the *de novo* case, leading to a broader range of observed effective selection values. In our model, transmission was assumed to be a neutral process, such that even lower fitness viruses could potentially contribute to the founding population; further restrictions on the modelled transmission process could potentially reduce the variance towards that of the *de novo* case. To give one example, if founding infection was difficult, and the small number of viruses that achieve this were the survivors of a competition between a much larger number of viruses, the range of fitnesses of viruses in the founder population could be reduced.

In our simulations of transmitted standing variation, mutational load had a large effect on the fate of the beneficial mutation. Where mutational load was neglected, a variant reached fixation in all of our simulations for which the strength of selection s≥1, but fixation was never achieved for cases with s≤0.5 (Fig. 6B). Mutational load affected this in both directions. On the one hand, under mutational load, a variant with *s* = 0.25 was sometimes sufficient to achieve fixation, the initial background of a variant aiding its evolutionary progress. On the other, even very strong positive selection (*s* = 5) was insufficient to guarantee the fixation of a variant.

Under our simulation conditions, the loss of the beneficial variant was exceptionally unlikely to occur via genetic drift; a beneficial mutation in a rapidly expanding population is expected to grow rapidly in frequency. As such, in the absence of mutational load, loss of the beneficial variant was never observed. In contrast, under mutational load beneficial mutations were frequently lost from the population. Despite its inherent advantage, loss of the beneficial variant was observed in multiple simulations, occurring in 23 per cent of cases for which *s* = 0.25 and a little over 1 per cent of simulations for which *s* = 1 (Fig. 6C). Data shown were generated using the WF-CM model; the Wright-Fisher model led to mutational load having a greater impact on the probability of the fixation or loss of a variant (Supplementary Fig. S7).

## 4. Discussion

Using a novel evolutionary model of within-host viral infection, we have here considered the evolutionary implications of mutational load upon the evolution of beneficial variants in a within-host population based upon biologically realistic evolutionary parameters. Previous studies have considered the effect of mutational load in global viral populations (Pybus et al. 2007; Illingworth and Mustonen 2012; Koelle and Rasmussen 2015); here we have shown that it has a considerable impact upon the within-host evolution of a virus. Using parameters which reflect an influenza infection, mutational load was found to decrease the effective advantage conferred on a virus by a *de novo* beneficial variant by ∼10 per cent. Under mutational load, the effective selection is highly stochastic, with a transmitted variant potentially having a more than five-fold uncertainty in its effective selective effect. Even where strongly beneficial variants act for phenotypes such as immune escape or drug resistance, stochastic effects have a considerable influence on evolution.

Our result has implications for studies which have sought to estimate the magnitude of selection acting upon variants in viral populations. Given data from a single replicate, the inferred fitness effect of a beneficial variant is intrinsically uncertain, with a bias towards a lower selective effect. In so far as mutational load consists of a large number of mutations spread across a genome at low allele frequencies, the direct assessment of its effect via genome sequencing is likely to be very challenging using current sequencing technology. Studies which have used sequence data from within-host infections to evaluate the fitness of beneficial mutations are therefore likely to have underestimated the benefit of such mutations (Fonville et al. 2013; Foll et al. 2014; Illingworth, Fischer, and Mustonen 2014; Illingworth 2015). We note that this effect is not constrained to population genetic approaches to selection. Viral competition experiments (Marée et al. 2000) and deep mutational scanning (Thyagarajan and Bloom 2014) each involve a comparison of the prevalence or growth rate of viruses, which either have or do not have a specific variant. Where such experiments involve a process of error-prone viral replication, mutational load will have an effect on the results; inferred fitnesses obtained in this way will in the mean underestimate true fitness differences between variants.

Our result also has implications for studies which have estimated the probability of the emergence or fixation of variants during the course of a single infection. For example, in evaluating the potential for a zoonotic infection to become increasingly adapted to a human host, the likelihood that new adaptive variants become established is key (Russell et al. 2012; Reperant et al. 2014). In so far as studies of this phenomenon have not accounted for mutational load, they have likely overestimated the extent to which the gain of beneficial mutations might be expected to occur. A recent study has noted an absence of strongly adaptive mutations in natural within-host influenza infection (McCrone et al. 2018); here we have shown that, as the effects of mutational load work against the onset of new mutations, adaptation to a new host requires stronger selection than has previously been appreciated.

### 4.1 Application to other viruses

Although our simulation model was parameterised using data from studies of influenza virus, the results we obtain are likely to be easily translatable to other RNA viruses causing acute infectious illness, according to the mutation rate, distribution of fitness effects, and demography of a given viral infection. A key determinant of the effect of mutational load is the basic rate of viral mutation. Estimates of mutation rates for RNA viruses span a range of slightly greater than an order of magnitude (Sanjuan et al. 2010); new experimental techniques may identify such rates with greater precision. Distributions of fitness effects have been characterised for only a small number of RNA viruses, but show a qualitatively common pattern, combining a largely deleterious unimodal distribution with a proportion of lethal mutations (Acevedo, Brodsky, and Andino 2014; Visher et al. 2016). Changes in the overall size of the viral population in our model caused relatively smaller changes in the influence of mutational load, albeit with a greater effect of mutational load at larger population sizes. A longer period of infection would affect the results obtained in a relatively straightforward manner; weaker magnitudes of selection can produce equivalent effects upon a population given longer periods of time in which to act.

### 4.2 Evolutionary modelling

We have here generated results from a standard Wright-Fisher population model, and from a new WF-CM, in which the timing of mutations was modelled to follow a uniform distribution. As we have shown, the two models may be derived from two limiting assumptions about the rate to which viral RNA within a cell is repeatedly copied during the process of viral replication. Regarding influenza virus, we note that the potential theoretically exists for more than one round of RNA replication to occur within a cell. During infection, positive-strand cRNA is produced by the infecting ribonucleoprotein (vRNP), which is copied to negative strand vRNA; this RNA is encapsidated into new vRNPs (Heldt, Frensing, and Reichl 2012). The vRNPs produced via replication outnumber those in the infecting viruses, and have the capacity to produce further cRNA. However, measurements of cRNA in cells infected at high MOI show the production of viral RNA to spike early in infection (Smith and Hay 1982), while direct measurements of cRNA levels in the cell show that their initially rapid increase is followed by a levelling off or slowed increase in concentration (Kawakami et al. 2011). These results do not support the idea that cRNA is substantially produced by vRNPs other than those which initiated infection; if this is the case, the effect of mutational load during infection may be closer to the results derived from the standard Wright-Fisher model, rather than the more conservative WF-CM model. We note that the WF-CM model has broader potential application than the specific usage made here; non-uniform distributions of the timings of mutation, arising from other replication scenarios, could be considered.

With regard to parameters of our model, our approach has been to incorporate as much information as possible from experimental virological studies; this is most straightforward in the consideration of viral mutation rate and the fitness effects of mutations. An aim to replicate real infections has also motivated our consideration of viral demographics. A broad tradition of studies has modelled within-host populations in terms of changes in viral titre (Beauchemin and Handel, 2011). Such an approach allows a direct fitting to viral titre data (Handel, Longini, and Antia 2007; Saenz et al. 2010; Canini and Carrat 2011; Pawelek et al. 2012), albeit it does not allow an accounting for individual viruses. Our demographic model is based upon insights into the relative changes in population size described by these models, albeit that we consider an absolute number of viruses. An explicit population size can account for differences in the outcome of infection given different initial viral titres (Graham and Braciale 1997), a property which is not captured by models of cell-limited infection (Supplementary Fig. S8).

Previous models of explicit influenza demographics have taken different approaches to considering viral reproduction. For example, a previous modelling study (Russell et al. 2012) noted that, while a cell infected with the influenza virus generates ∼10^4^ new viral particles (Sidorenko and Reichl 2004), these viruses infect an average of twenty-two new cells (Baccam et al., 2006). Using the former figure to simulate a 10,000-fold increase in the viral population per generation led in this case to a peak viral population of 10^14^, although the inclusion of immune effects into this framework produces a lower peak titre (Reperant et al. 2014). Our approach differs from this in using the latter figure for the rate of viral replication, resulting in a peak population of between 5 × 10^6^ and 5 × 10^8^ viruses. In this respect our model assumes a picture of cellular equivalence; if in one generation a total of *v* viruses infect *c* cells, in the next generation, where 22*c* cells are infected, we assume that 22*v* viruses comprise the viral population. Our demographic model shows some correspondence with direct observations of infection, which describe peaks densities of between 10^5^ and 10^8^ RNA copies per ml (Pawelek et al. 2012). Combining this with the assumption that between 10^2^ and 10^3^ ml of sample exist within a host gives an estimated 10^7^ to 10^11^ particles within a host, or between 10^5^ and 10^9^ functional viruses if 1 per cent of viruses are assumed to be active (Wei et al. 2007). We note that further insights into the within-host biology of infection could refine the properties of our model.

## 5. Conclusions

We have here described a model evaluating how mutational load contributes to the within-host evolution of beneficial variants during within-host viral infection. Beneficial mutations are in the mean less likely to fix or establish in a population, albeit that their effective fitness may be strongly influenced by stochastic effects. In so far as possible, our model uses experimental data describing the parameters of viral evolution to produce a realistic model of within-host infection. The increasing ability to characterise fitness landscapes on a high-throughput basis, combined with new approaches in population genetic theory, make it possible to achieve far more realistic assessments of how a viral population might be expected to behave during the course of an infection, with multiple potential applications in the quest to better understand viral evolution.

Our work grants an increased understanding of stochastic and deterministic effects in acute RNA viral infection. A recent study of within-host influenza infection observed few large changes in allele frequency during the course of untreated within-host influenza infection, concluding that within-host evolution is dominated by stochastic effects, such as genetic drift, rather than by the influence of selection (McCrone et al. 2018). Our results show that the strength of selection required to produce adaptive change is greater than previously appreciated; even where selection is strong, adaptation occurs against a generally hostile background of mutational load. Furthermore, we have shown that stochastic behaviour in a population does not always arise from the dominance of genetic drift over selection. In a large population, in which strong selection drives adaptation, and where genetic drift has little effect, stochastic effects induced by mutational load have a significant effect upon the outcome of within-host viral evolution.

## Supplementary Material

Supplementary Data
