# Supplementary material for: Mutational load causes stochastic evolutionary outcomes in acute RNA viral infection
Source: Virus Evol. 2019 Apr 22;5(1):vez008. doi: 10.1093/ve/vez008 (PMC6476161; doi:10.1093/ve/vez008)
Supplement: Supplementary Data [file vez008_supp.pdf]

Mutational load causes stochastic evolutionary outcomes in acute RNA viral infection

Lei Zhao[1], Ali Abassi[2], and Christopher J. R. Illingworth[1,2,*]

[1]Department of Genetics, University of Cambridge, Cambridge, UK
[2]Department of Applied Mathematics and Theoretical Physics, University of Cambridge, Cambridge, UK
[*]chris.illingworth@gen.cam.ac.uk

Mathematical Appendix

Derivation of the formula for effective selection

In the main text, we present a formula for the effective selection acting upon an allele, calculated in terms of the frequency of the allele at subsequent time-points. This was derived from the equation for a change in allele frequency in the WF-CM model. In this model, the change in an allele frequency can be calculated as

$$\begin{aligned}
q(t_{k+1}) &= \frac{(1+s_{\text{eff}})\left[q(t_k)(1-\mu)+\frac{\mu}{3}(1-q(t_k))\mathbb{E}\left(1-\tau^{(i)}\right)+\mu q(t_k)\mathbb{E}\left(\tau^{(o)}\right)\right]}{1+s_{\text{eff}}\left[q(t_k)(1-\mu)+\frac{\mu}{3}(1-q(t_k))\mathbb{E}\left(1-\tau^{(i)}\right)+\mu q(t_k)\mathbb{E}\left(\tau^{(o)}\right)\right]} \\
&= \frac{(1+s_{\text{eff}})\left[q(t_k)(1-\mu)+\frac{\mu}{6}(1-q(t_k))+\frac{\mu}{2}q(t_k)\right]}{1+s_{\text{eff}}\left[q(t_k)(1-\mu)+\frac{\mu}{6}(1-q(t_k))+\frac{\mu}{2}q(t_k)\right]} \\
&= \frac{(1+s_{\text{eff}})\left[q(t_k)(1-\frac{\mu}{2})+\frac{\mu}{6}(1-q(t_k))\right]}{1+s_{\text{eff}}\left[q(t_k)(1-\frac{\mu}{2})+\frac{\mu}{6}(1-q(t_k))\right]},
\end{aligned} \tag{1}$$

where $q(t_k)$ represents the frequency of the beneficial variant at generation t_k, and $\mathbb{E}(\cdot)$ denotes the expectation of a random variable. Given the focal site will change during generation t_k, $\tau^{(o)}$ and $\tau^{(i)}$ represent the times when a focal beneficial mutation carrier mutates to the wild type, and when a wild type carrier achieves the focal beneficial mutation, respectively. In Equation 1, $(1+s_{\text{eff}})q(t_k)(1-\mu)$ is the fraction (without rescaling) of offspring carrying the beneficial variants given their parents do not undertake mutations at the focal site during generation t_k, $\mu(1+s_{\text{eff}})q(t_k)\mathbb{E}\left(\tau^{(o)}\right)$ is the non-rescaling fraction of beneficially mutated offspring given their parents mutate from mutated type to wild type during generation t_k, and $\frac{\mu}{3}(1+s_{\text{eff}})(1-q(t_k))\mathbb{E}\left(1-\tau^{(i)}\right)$ is the corresponding fraction of beneficially mutated offspring contributed by the individuals who mutate from wild type to mutated state in generation t_k.

From this derivation we obtain the formula used in the main text for inference of Wright-Fisher model with continuous mutations,

$$s_{\text{eff}} = \frac{q(t_{k+1})-q(t_k)(1-\frac{\mu}{2})-\frac{\mu}{6}(1-q(t_k))}{(1-q(t_{k+1}))\left[q(t_k)(1-\frac{\mu}{2})+\frac{\mu}{6}(1-q(t_k))\right]}. \tag{2}$$

If $\mathbb{E}\left(\tau^{(i)}\right)=\mathbb{E}\left(\tau^{(o)}\right)=0$, which corresponds to the instantaneous mutations at the beginning of each generation, Equation (1) will degenerate to describe the original Wright-Fisher model,

$$q(t_{k+1}) = \frac{(1+s_{\text{eff}})\left[q(t_k)(1-\mu)+\frac{\mu}{3}(1-q(t_k))\right]}{1+s_{\text{eff}}\left[q(t_k)(1-\mu)+\frac{\mu}{3}(1-q(t_k))\right]}, \tag{3}$$

and the corresponding equation to infer the effective selection of the original Wright-Fisher model is as follows,

$$s_{\text{eff}} = \frac{q(t_{k+1})-q(t_k)(1-\mu)-\frac{\mu}{3}(1-q(t_k))}{(1-q(t_{k+1}))\left[q(t_k)(1-\mu)+\frac{\mu}{3}(1-q(t_k))\right]}. \tag{4}$$

Derivation of the uniform time distribution of mutations in the WF-CM model

Within our continuous-mutation Wright-Fisher model we assume that viruses accumulate mutations over time in a uniform manner. We here discuss this assumption in the context of influenza infection.

A highly detailed model of the intracellular infection process has been given elsewhere[?]. We here consider a similar model, albeit one including a number of simplifying assumptions. Heldt et al., show that the rate at which viruses are released by the cell increases gradually from 3 hours post adsorption of the first virus until the end of the period of cellular infection. We here approximate this process, assuming that viral production occurs at a constant rate over time. Next, where Heldt et al. model a complex process of RNA replication and protein production we assume that proteins are produced and released instantaneously, being translated from the pool of viral RNA that exists within a cell at an given time. Finally, we model the replication of viral RNA as a simple branching process.

In our highly simplified model, each strand of RNA replicates at a constant rate within the cell, beginning with a single copy of each strand. We assume that the timing of strand replication occurs according to an exponential distribution, such that the mean time for one of n strands to replicate is given by $1/n$; ignoring deviation from this timescale implies that replication round r occurs at the time

$$t_r = \sum_{i=1}^{r} \frac{1}{i} \tag{5}$$

We note that after r rounds of replication there are $r+1$ copies of each strand.

We now consider the mean number of replication events undergone by a strand in the population after r replications, which we denote by m_r. At any given time, the total number of replication events to have occurred in the population as a whole is given by $m_r^{tot} = (r+1)m_r$. During the next replication event, a strand is chosen for replication, which produces a copy of itself that has undergone one additional replication. The new population will contain $r+2$ strands. Therefore

$$\begin{aligned} m_0 &= 0 \\ m_{r+1} &= \frac{(r+1)m_r + m_r + 1}{r+2} \\ &= m_r + \frac{1}{r+2} \end{aligned} \tag{6}$$

producing an increase of $1/(r+2)$ in m_r in the time interval $1/(r+1)$ between t_r and t_{r+1}. It may be straightforwardly observed the mean number of replications undergone by a strand in the population increases roughly linearly over time.

Thus, considering the proteins produced by a mean strand in the cell, it will be noted that initially, these proteins will contain no mutations relative to the infecting virus. As time goes by, the strand will accumulate mutations linearly over time through replication error, the same accumulation of mutations being seen in the proteins produced by translation. Thus our simplified model of replication leads to a WF-CM model which has a uniform distribution in the timings of mutations.

Numerical approximations in the simulation

In order to reduce the computational time required by our simulation, a series of approximations were applied as the population size became larger. Within our calculation, the population is divided into bins of viruses with roughly equal fitness. We suppose that there are N viruses in a bin. In each generation, to account for mutation, we first calculated n, the number of mutations incurred by viruses in that bin, as a Poisson distributed random variable with parameter $N\mu L$, where μ is the viral mutation rate per base, and L is the length of the viral genome. The next step was then dependent upon the viral population size. At small population sizes (less than 5×10^5), viruses were then uniformly chosen from the set of N viruses, assigning each mutation to a virus in turn. At population sizes between 5×10^5 and 5×10^6, a binomial distribution was used to calculate the expected proportion p_i of viruses affected by i mutations. In so far as the probability of a specific virus being affected by a given mutation is $1/N$, these values were calculated as

$$p_i = \binom{n}{i} \left(\frac{1}{N}\right)^i \left(1 - \frac{1}{N}\right)^{n-i} \tag{7}$$

The number of viruses receiving i mutations, a_i, was then calculated as a sample from a multinomial distribution.

$$P(\{a_0, a_1, \ldots\}) = \frac{n!}{\prod_i a_i!} \prod_i p_i^{a_i} \tag{8}$$

A cap of seven mutations per generation was applied to each virus. At population sizes between 5×10^6 and 10^7 the multinomial was further approximated by a series of Poisson distributed random variables, with a_i being calculated as a sample from a Poisson distribution with rate Np_i, for $i \geq 1$. Finally a_0 was calculated as $N - \sum_{i \geq 1} a_i$. At population sizes greater than 10^7 this step was further approximated, a_i being calculated as the expectation of the Poisson distribution with rate Np_i. Collectively these approximations sped up the implementation of our code.

Supplementary Figures

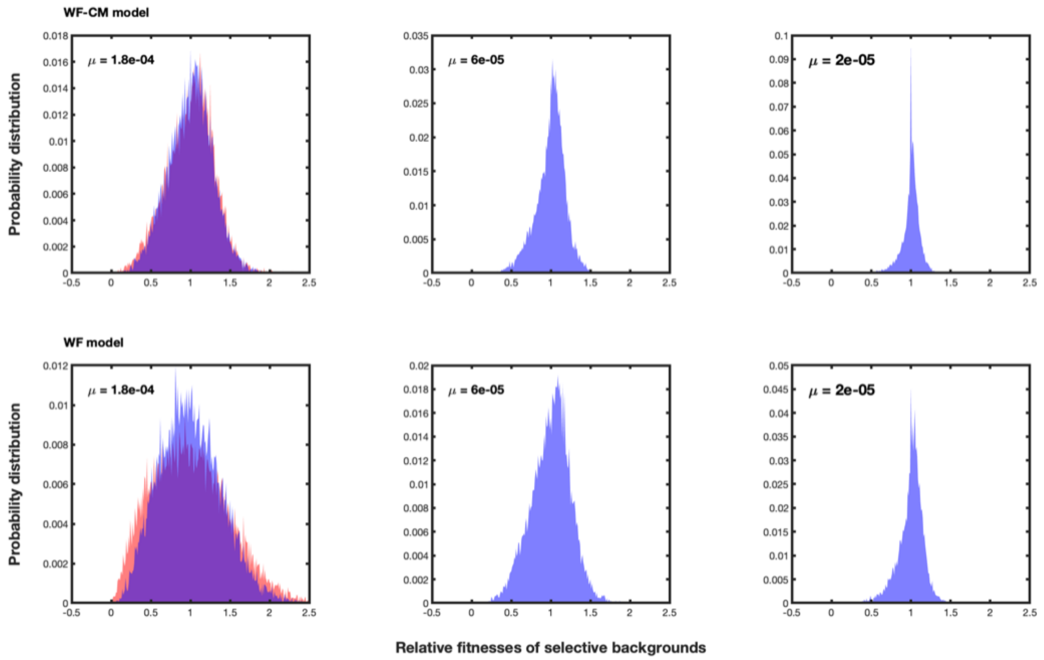

Figure S1. Equilibrium distribution of relative viral fitnesses immediately following transmission of five viral particles. A fitness of 1 corresponds to the mean fitness of the transmitted population. Blue distributions show results under a multiplicative model of selection; red shows results including the effects of epistasis.

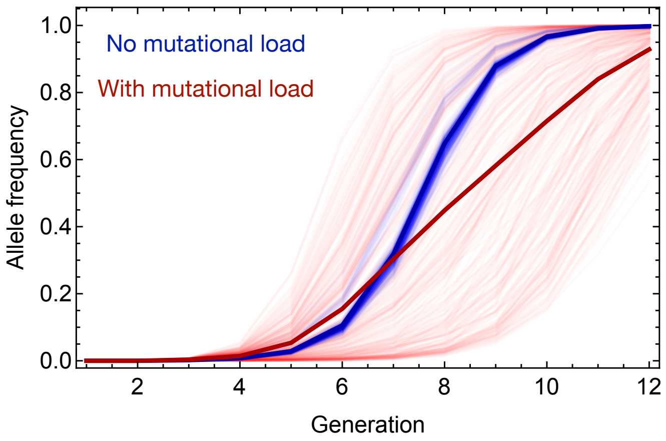

Figure S2. Example trajectories for the beneficial variant under the Wright-Fisher model with (red) and without (blue) mutational load. Selection here is equal to 3. Bold lines indicate mean trajectories. Trajectories shown here are those in which the beneficial variant is first observed in the third generation of the simulation.

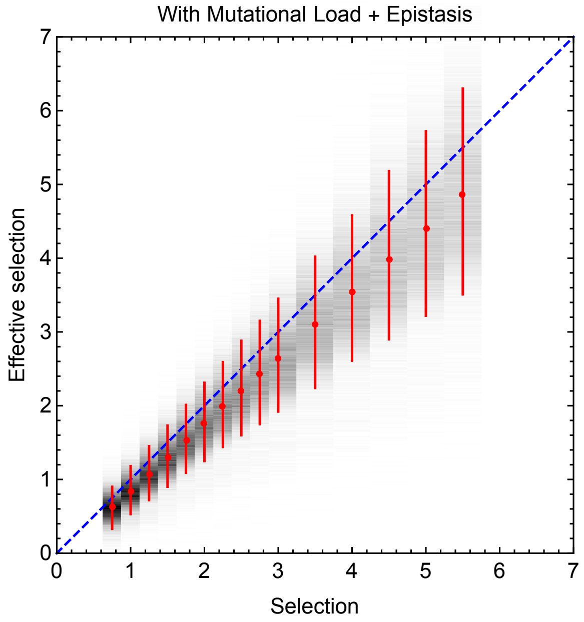

Figure S3. Effective selection coefficients from simulations conducted using the WF-CM model under default parameters, including mutational load with epistasis. Vertical red bars show 90% confidence intervals for the effective selection. Gray shading represents the distribution of inferred effective selection values. The blue dotted line shows equivalence between the true and effective selection coefficients.

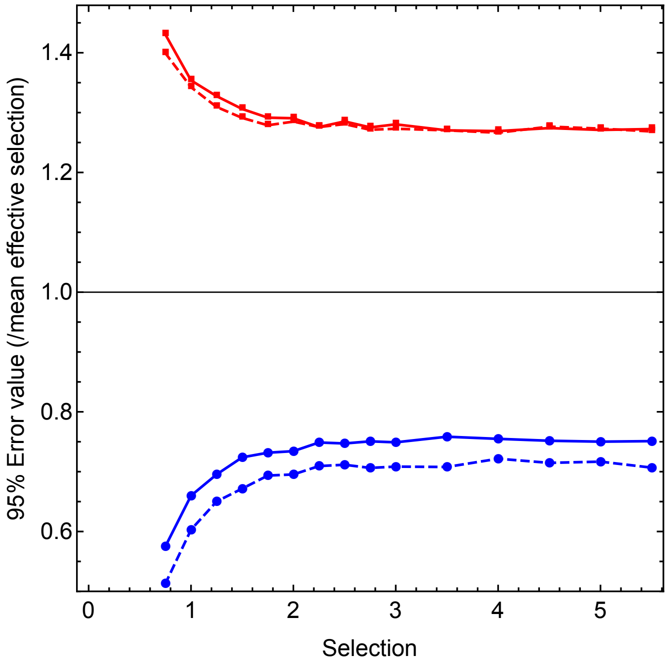

Figure S4. Upper (red) and lower (blue) one-tailed 95% confidence values for the effective selection of a beneficial variant in the case of the *de novo* emergence of a beneficial variant in a population under our default model parameters. Confidence values mark the top and bottom 5% of effective selection coefficients as a fraction of the mean and are shown for simulations run with mutational load and with (dashed lines) and without (solid lines) epistasis. Error bars tend to approximately $\pm$ 25% of the mean effective selection.

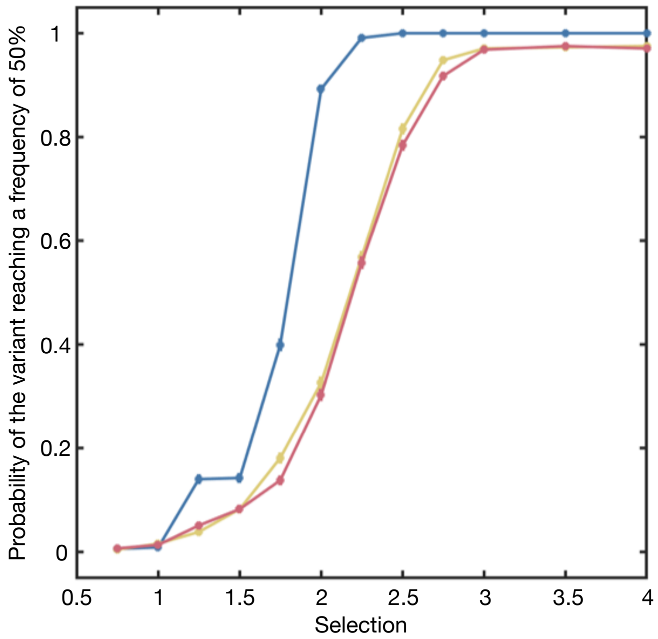

Figure S5. Probability that the beneficial variant will rise to a frequency of 50% or greater during the course of an infection. Data are shown from simulations that exclude the effects of mutational load (blue) or include it, either under an additive model of selection (yellow) or under a model incorporating negative epistasis (red). Simulations were conducted with an initial population bottleneck of 5 and a mutation rate of 1.8×10^{-4} per base per generation. Error bars show estimated 95% confidence intervals in the reported mean values. Data are shown here for the Wright-Fisher model.

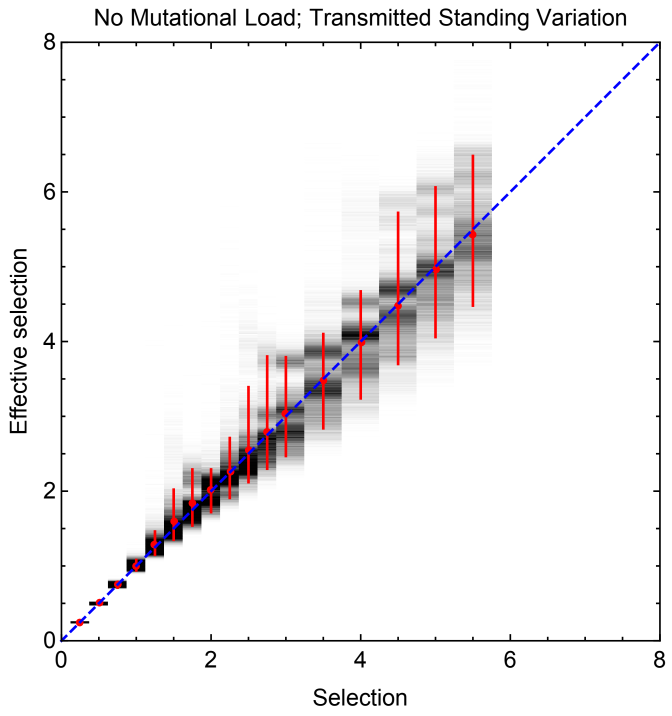

Figure S6. Effective selection coefficients inferred from simulations conducted using the WF-CM model for the case in which a single transmitted virus carries the beneficial allele, and in which mutational load was not included in the simulation. Vertical red bars show 90% confidence intervals for the effective selection. Gray shading represents the distribution of inferred effective selection values. The blue dotted line shows equivalence between the true and effective selection coefficients.

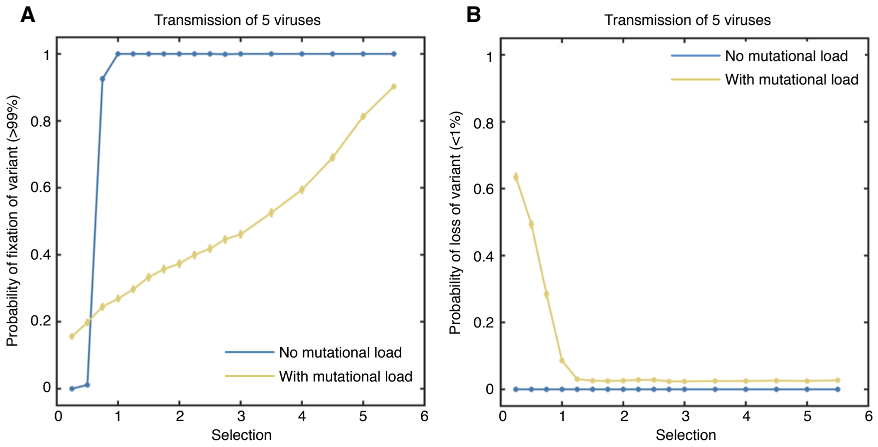

Figure S7. Effect of mutational load upon a transmitted variant. Simulations were initiated with one out of five viruses carrying the beneficial allele. Data are shown here for the Wright-Fisher model. **A.** Probability that the beneficial variant will reach fixation during the course of an infection. Results are shown for simulations in the absence (blue) or presence (yellow) of mutational load. **B.** Probability that a variant will die out during the course of infection. Error bars show the extent of variation across 10,000 simulations for each point. The probability of death does not tend to zero due to the possibility of all viruses to be lethally mutated in the first generation; this event does not occur in the WF-CM model

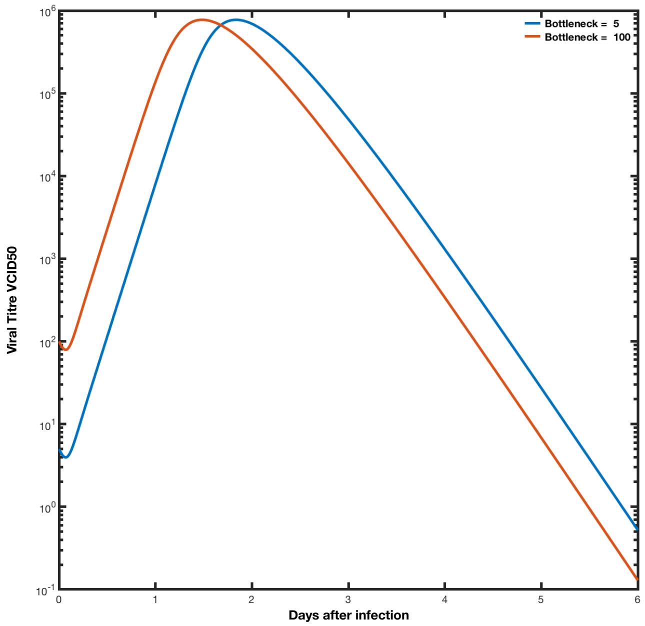

Figure S8. Change in relative viral load under the model described by Beauchemin and Handel (2011) given initial populations that differ by two orders of magnitude. In a model where infection is limited by a fixed number of uninfected cells, the peak number of viruses in the system is largely independent of the number of particles founding infection.
